# PD-1 and LAG-3 Checkpoint Blockade: Potential Avenues for Therapy in B-Cell Lymphoma

**DOI:** 10.3390/cells10051152

**Published:** 2021-05-10

**Authors:** Joshua W. D. Tobin, Karolina Bednarska, Ashlea Campbell, Colm Keane

**Affiliations:** 1 Mater Research Institute, University of Queensland, Brisbane, QLD 4102, Australia; jwd.tobin@gmail.com (J.W.D.T.); karolina.bednarska@mater.uq.edu.au (K.B.); 2 Department of Haematology, Princess Alexandra Hospital, Brisbane, QLD 4102, Australia; ashlea.k.campbell@gmail.com

**Keywords:** immune checkpoint blockade, PD-1, LAG-3, Hodgkin lymphoma, non-Hodgkin lymphoma, follicular lymphoma, diffuse large B cell lymphoma

## Abstract

The dependence of cancer on an immunotolerant tumor microenvironment (TME) is well established. Immunotherapies that overcome tumor-induced immune suppression have been central to recent advancements in oncology. This is highlighted by the success of agents that interrupt PD-1 mediated immune suppression in a range of cancers. However, while PD-1 blockade has been paradigm-shifting in many malignancies, the majority of cancers show high rates of primary resistance to this approach. This has led to a rapid expansion in therapeutic targeting of other immune checkpoint molecules to provide combination immune checkpoint blockade (ICB), with one such promising approach is blockade of Lymphocyte Activation Gene 3 (LAG-3). Clinically, lymphoproliferative disorders show a wide spectrum of responses to ICB. Specific subtypes including classical Hodgkin lymphoma have demonstrated striking efficacy with anti-PD-1 therapy. Conversely, early trials of ICB have been relatively disappointing in common subtypes of Non-Hodgkin lymphoma. In this review, we describe the TME of common lymphoma subtypes with an emphasis on the role of prominent immune checkpoint molecules PD-1 and LAG3. We will also discuss current clinical evidence for ICB in lymphoma and highlight key areas for further investigation where synergistic dual checkpoint blockade of LAG-3 and PD-1 could be used to overcome ICB resistance.

## 1. Introduction

Inhibitory checkpoint receptors play a critical role in immune homeostasis. In health, the expression of checkpoint receptors is upregulated following the activation of antigen specific T-cells to temper the pro-inflammatory response. However, upon prolonged activation with a persisting antigen, such as chronic viral infections or in cancer, checkpoint expression is maintained, and effector T-cells enter a state of ‘exhaustion’ [1,2]. Exhausted T-cells demonstrate a progressively reduced proliferative capacity and the loss of effector T-cell functions including the production of inflammatory cytokines and degranulation [3,4]. Accordingly, there has been a rapid expansion in therapeutic targeting of these checkpoint receptors to reinvigorate the effector functions of exhausted T-cells.

Therapeutic immune checkpoint blockade (ICB) of Programmed Death-1 (PD-1) receptor has shown remarkable efficacy in restoring effector T-cell function in malignancy and consequent clinical trials have shown unprecedented therapeutic gains in many solid tumors including melanoma, non-small cell lung cancers (NSCLC), and renal cell carcinoma [5,6,7]. Unfortunately, trials of PD-1 blockade in lymphoma have been less successful and clinical responses have been limited to a proportion of patients with Hodgkin lymphoma and rare Non-Hodgkin Lymphoma (NHL) subtypes. The reasons for the sub-optimal efficacy of these agents in lymphoma remain unclear and are an area of active research.

Nevertheless, the promising anti-tumor activity of these agents in a narrow range of lymphoma subsets has prompted continued interest in the development of newer checkpoint inhibitors and the employment of rational combinations of ICB agents to overcome T-cell exhaustion in lymphoproliferative diseases (LPDs). In this review we will discuss PD-1 and Lymphocyte Activation Gene 3 (LAG-3)—a promising candidate for dual checkpoint blockade therapy. We will summarize and compare pre-clinical studies exploring checkpoint molecule expression in a range of common lymphoma subtypes and summarize current clinical studies with ICB.

## 2. Programmed Death-1 Checkpoint Axis

**Figure 1:**
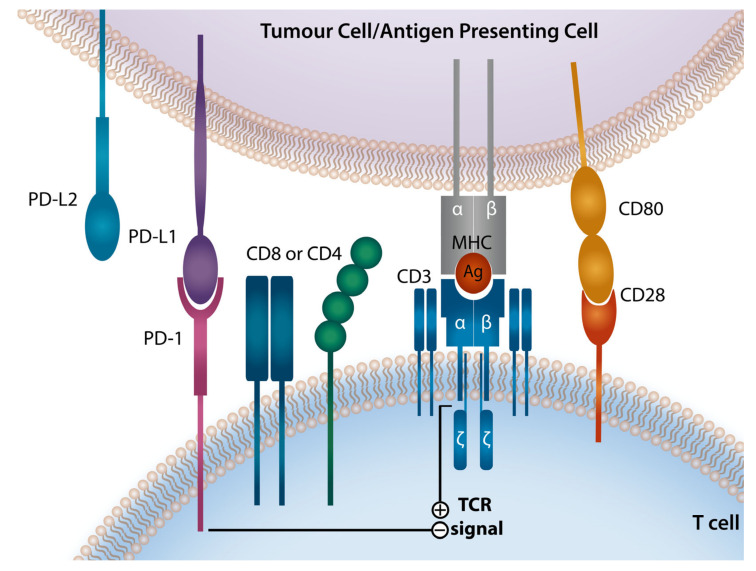
Programmed Death-1 Signaling Axis The PD-1/PD-L1/PD-L2 checkpoint pathway renders tumor cells resistant to T-cell immune attack. Ligation of T-cell PD-1 by PD-L1 or PD-L2 which are present on tumor cells or antigen presenting cell. PD-1 ligation dephosphorylates multiple members of the TCR signaling pathway attenuating TCR and CD28 signaling and promoting T-cell anergy and functional exhaustion. PD-1, programmed death-1; PD-L1, PD ligand-1; PD-L2, PD ligand-2; MHC, major histocompatibility complex; TCR, T-cell receptor.

The PD-1 receptor, also known as CD279, was discovered in 1992 [8]. It is a cell surface receptor with inducible expression on a range of immune cells including T-cells, NK cells, B cells, monocytes, and dendritic cells [9,10,11,12]. PD-1 and its cognate ligands, Programmed Death Ligand 1 (PD-L1) and 2 (PD-L2), are known to play a critical role in the regulation of T-cell function (Figure 1). PD-1 engagement triggers a signaling cascade that results in TCR signal attenuation that inhibits T-cell activation, proliferation, and cytokine production. PD-1 expression rapidly declines with antigenic clearance; however, antigen persistence leads to ongoing PD-1 expression and eventual T-cell exhaustion [13,14,15,16]. PD-1 expression on T-cells is upregulated by soluble inflammatory mediators such as IL-2, IL-7, IL-15, IL-21, TLRs and interferons (IFNs) [17]. Of note, PD-1 is also expressed on B cells and appears to inhibit BCR-mediated activation, proliferation, and IL-6 production [18,19].

PD-1 has two known ligands, PD-L1 (CD274 or B7-H1) and PD-L2 (CD273 or B7-DC), with varying expression patterns. PD-L1 expression can be detected on hematopoietic cells including T-cells, B cells, macrophages, dendritic cells (DCs), and mast cells, as well as non-hematopoietic healthy tissue cells [20]. Expression of PD-L2 is much more restricted and it is observed on macrophages, DCs, and mast cells [20,21]. The PD-1 ligands can be expressed on cell surfaces upon exposure to inflammatory cytokines, in particular interferons (INFs) [22,23,24,25]. Binding of IFN-γ to its receptor triggers a signaling cascade mediated via the Janus kinase (JAK)-signal transducer and activator of transcription (STAT) pathway, that results in increased expression of a range of transcription factors, known as the interferon-responsive factors (IRFs) [26]. Of those factors, IRF-1 has been shown to play a central role in the IFN-γ-mediated induction of PD-L1 and to a lesser extent PD-L2 [11,20,27]. In contrast, IL-4 and GM-CSF are the strongest stimuli inducing PD-L2 expression [11,14,28,29]. PD-L1-mediated signaling can further inhibit T-cell activation by competitively binding CD80, a ligand for a co-stimulatory receptor CD28 [27].

Malignant cells often exploit the PD-1/PD-L1/PD-L2 pathway, by expressing checkpoint ligands on their surface, to evade anti-tumor responses and thrive in the tumor microenvironment (TME) [12]. Hence, disruption of PD-1/PD-L1/PD-L2 signaling with PD-1 blocking monoclonal antibodies has become an attractive strategy to harness the immune effector cells anti-tumor potential in lymphoma as well as solid organ malignancy [28,29,30].

The employment of anti-PD-1 ICB has been a paradigm shift in a variety of solid malignancies. In particular anti-PD-1 therapy of metastatic melanoma has demonstrated marked improvement in outcomes, with 35–40% of patients now obtaining an objective partial or complete response with a median overall survival (OS) of approximately 3 years [31,32]. In lymphoma there are multiple ongoing clinical trials examining the effectiveness of PD-1 blockade, and regulatory approval has been granted for anti-PD-1 therapy in primary mediastinal B-cell lymphoma (PMBCL) and classical Hodgkin Lymphoma (cHL) in the relapsed/refractory (R/R) setting.

## 3. LAG-3 Checkpoint Axis

LAG-3 is a transmembrane protein that acts as an immune checkpoint and was first described in 1990 [33]. LAG-3 is expressed early in T-cell activation and reaches a peak in 24–48 h. LAG-3 shares significant homology to CD4 but binds to major histocompatibility (MHC) Class II (MHC-II) with an affinity approximately 100 times that of CD4 [34]. It is found on activated CD4^+^ and CD8^+^ T-cells, CD4^+^ regulatory T-cells (T_REG_), NK-cells and immunosuppressive plasma cells [35,36]. In LAG-3 knock-out (KO) animal models there is extensive T-cell over-expansion in response to infection [37]. In particular, induction of a superantigen or immunization with specific antigens can lead to overwhelming response of LAG-3 KO T-cells. High levels of LAG-3 on T-cells correlates with reduced ability to clear and control viruses such as Human Immunodeficiency Virus (HIV) [38,39].

**Figure 2:**
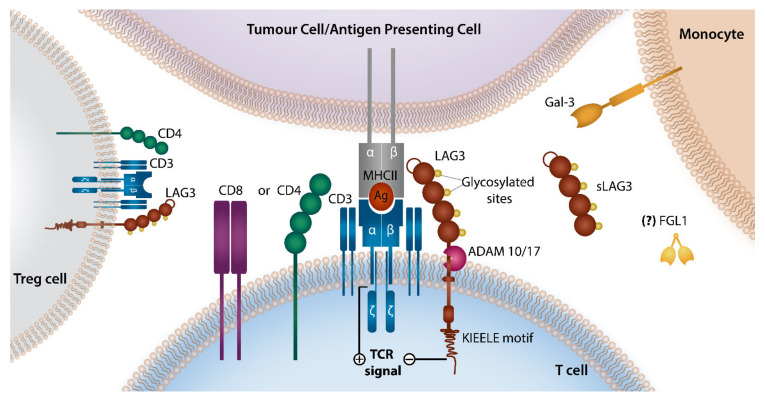
LAG-3 Signaling Axis LAG-3 and CD4 receptors share a similar structure consisting of four extracellular Ig-like domains, except for an additional 30 amino acid loop present on LAG-3 that ensures its greater binding affinity to MHC-II ligand and longer connecting peptide between the last Ig domain and the transmembrane region making it more susceptible to cleavage by ADAMs. The interaction between LAG-3 and MHC-II (expressed by tumor or antigen presenting cells) triggers inhibitory signaling that suppresses T-cell function. Alternative LAG3 ligands such as Gal-3, FGL1, and CLEC4G are also described but their role in lymphoma remains to be elucidated. LAG-3, Leukocyte Activation Gene-3; MHC-II, Major Histocompatibility Complex class II; ADAM, a disintegrin and metallopreoteinase domain-containing protein Gal3, galectin 3; FGL1, fibrinogen-like protein.

However, a number of new ligands for LAG-3 have been recently described that may help to provide insights into the impacts of LAG-3 on immune responses in cancer (Figure 2). C-Type Lectin Domain Family 4 Member G (CLEC4G or LSECtin) is one such ligand that can be found in normal liver but has also been found on melanoma cells [40]. Galectin-3 has also been described as a LAG-3 ligand that specifically inhibits CD8^+^ T-cells and alters plasmacytoid dendritic cell responses in pancreatic cancer models [41].

Fibrinogen-like protein 1 (FGL1) is an important newly discovered ligand of LAG-3 that causes significant reduction in anti-tumoral immunity [42]. Interestingly FGL1 does not compete with MHC-II for LAG-3 binding. FGL1 expression is found in a number of tumors and is associated with poor outcome. It is possible that current anti-LAG-3 antibodies may also need to target LAG-3-FGL1 interactions to fully reactivate exhausted T-cells. In addition, high level of circulating FGL1 is associated with inferior outcomes in NSCLC and melanoma patients treated with anti-PD-1 therapy [42]. It should be noted that the understanding of the importance of all these ligands remains at an early stage and there is limited data to inform on the importance of these ligands in lymphoma.

The cell surface expression of LAG-3 is controlled by two known mechanisms. On T-cell activation, LAG-3 is rapidly translocated from lysosomes to the cell surface. In the resting state, LAG-3 is degraded in these lysosomal areas. Alternatively, LAG-3 cell surface expression is controlled by cleavage of the molecule by ADAM10 and ADAM17. Interestingly this cleaved LAG-3 does not appear to compete with cell surface LAG-3 for MHC-II binding. However, cleavage of LAG-3 does appear critical as the presence of non-cleavable mutants leads to reduced T-cell function [43].

Work to date has focused in the main on how LAG-3 induces immunosuppression in effector T-cells. With regards to lymphoma, the small number of studies performed to date indicate the likely important contribution of LAG-3 to immune suppression within the TME and early evidence points to particular importance in cHL and PMBCL. However, the strong association of LAG-3 with T_REG_ cells and its constitutive expression on these cells indicates that there is likely to be a number of factors that induce LAG-3 associated immunosuppression. It appears in some tumors, such as cHL, the effect of LAG-3 on T_REG_ cells may have a significant impact [44].

LAG-3 is frequently co-expressed with other immune checkpoint markers in exhausted T-cells. Murine models indicate that combined blockade of the PD-1 axis and LAG-3 enhances rejection of multiple tumor types, even when single agent blockade of anti-PD-1 therapy was ineffective [45].

IMP321 (a sLAG-3-Ig agent) was the first LAG-3 based molecule in clinical trials and has been combined with vaccination and chemotherapy strategies to try and elicit anti-tumor responses as an apparent immune agonist [46,47]. Ongoing phase I clinical studies of LAG-3, predominantly in solid tumors, show promising early results [48,49]. Interestingly, the addition of an anti-LAG-3 antibody to PD-1 blockade in patients who developed resistance to anti-PD-1 has resulted in second responses in solid tumors [48]. The recent development of bispecific antibodies (e.g., FS118, and NCT03440437) that can engage LAG-3 and PD-L1 presents a novel approach to immune checkpoint therapy. Early murine studies indicate an additional alternative mechanism of action (MOA) for this antibody whereby FS118 leads to enhanced shedding of LAG-3 from the T-cell surface which may indicate an enhanced ability to eradicate tumors more effectively [50].

## 4. Immune Checkpoint Molecules in Classical Hodgkin Lymphoma

### 4.1. Role of PD-1 Axis in Classical Hodgkin Lymphoma

CHL is a B-cell malignancy characterized by the scarce presence of neoplastic, Hodgkin and Reed–Sternberg (HRS) cells, embedded into the rich infiltrate of immune cells [51]. A hallmark of HRS cells is a high expression of PD-1 ligands on their surface. This is due to a high prevalence of 9p24.1 chromosomic alteration in almost all cases [51,52]. CHL patients harboring PD-L1/L2 alterations with high PD-L1 expression have been shown to have a significantly shorter progression-free survival (PFS) to standard chemotherapy [53]. However, patients with higher levels of PD-L1 expression driven by genetic alterations in 9p24.1 and intact expression of MHC-II had superior outcomes after PD-1 blockade [52].

As well as the near universal copy number gains or amplifications of 9p24.1, other mechanisms promote high PD-1 axis expression in cHL. The 9p24.1 region covers loci encoding PD-L1/L2 and JAK2. Amplification of JAK2 gene leads to increased protein expression and consequently enhanced JAK2/STAT signaling which, via modulation of the PD-1 ligands promoter activity, drives further transcription of PD-L1 and PD-L2 [51]. PD-L1 expression can be further increased by Activator Protein 1 (AP-1) signaling, as AP-1 responsive enhancer element is located in intron 1 of PD-L1, but not PD-L2 [15]. HRS cells display a constitutive activation of AP-1 components, c-Jun and JunB, that upon binding by the PD-L1 enhancer increase the PD-L1 promoter activity and in turn the PD-L1 expression [9,13,15,29]. Additionally, in 30–40% of cHL patients, malignant cells demonstrate latent infection with Epstein Barr virus (EBV) [54]. The EBV derived latent membrane protein 1 (LMP1) mimics CD40 and activates NF-κβ signaling which consequently induces PD-L1 expression in a NF-κβ-dependent manner. LMP1 is also known to mediate the up-regulation of PD-L1 through activation of other pathways, i.e., AP-1 and JAK/STAT [13,55,56,57]. Despite the frequency of EBV infection, cHL cases associated with EBV (EBV^POS^) have been reported to have the same degree of PD-L1/PD-L2 copy number alterations as non-EBV associated cases (EBV^NEG^) [13].

PD-L1 expression in cHL is also found on non-malignant, tumor-associated macrophages (TAMs). These PD-L1^+^ TAMs not only localize in the vicinity of PD-L1^+^ HRS cells but also interact with PD-1^+^CD4^+^ T-cells. This indicates that PD-L1^+^ TAMs, by engaging PD-1 on tumor-infiltrating lymphocytes (TILs) can inhibit the immune responses in cHL [58]. Several studies show that increased numbers of TAMs and a macrophage-related gene expression signature are associated with poor clinical outcomes in both first and subsequent lines of therapy emphasizing the prognostic significance of TAMs in cHL [59,60,61,62,63,64,65,66,67]. Both PD-L1 and PD-L2 expression can be induced in TAMs by exposure to IFNs, in particular, IFN-γ [68]. PD-L1 is regulated by the IFN-γ signaling that involves binding of nuclear IRF-1 to the PD-L1 promoter, PD-L2 is regulated by IFN-β and IFN-γ, through both STAT3 and IRF-1 binding to its promoter. PD-L2 expression is also induced on inflammatory macrophages by IL-4 [25]. A recent study by Arlt et al., confirms the capacity of HRS cells to attract monocytes and drive their differentiation to TAMs [69].

In summary, PD-1 ligands are upregulated via tumor-intrinsic mechanisms (i.e., 9p.24 copy gains, EBV mediated LMP1) on RSCs and tumor-extrinsic signaling to TAMs (i.e., interferons) in cHL to create a tolerogenic immune microenvironment. While the advent of PD-1 inhibitors has improved treatment outcomes in cHL, the MOAs of PD-1 blockade are incompletely understood. The unique sensitivity of cHL to PD-1 blockade therapies is in part explained by the tumor intrinsic genomic alterations of the malignant cells of cHL that overexpress PD-L1 and PD-L2 ligands which, by engaging with PD-1 molecules on T-cells, drive their exhaustion [70,71]. Despite the initial sensitivity to PD-1 blockade, most patients eventually experience a relapse despite the persistence of these same tumor-intrinsic abnormalities suggesting alternate mechanisms exist that contribute to the development of the permissive TME in cHL.

### 4.2. Role of LAG-3 in Classical Hodgkin Lymphoma

**Figure 3:**
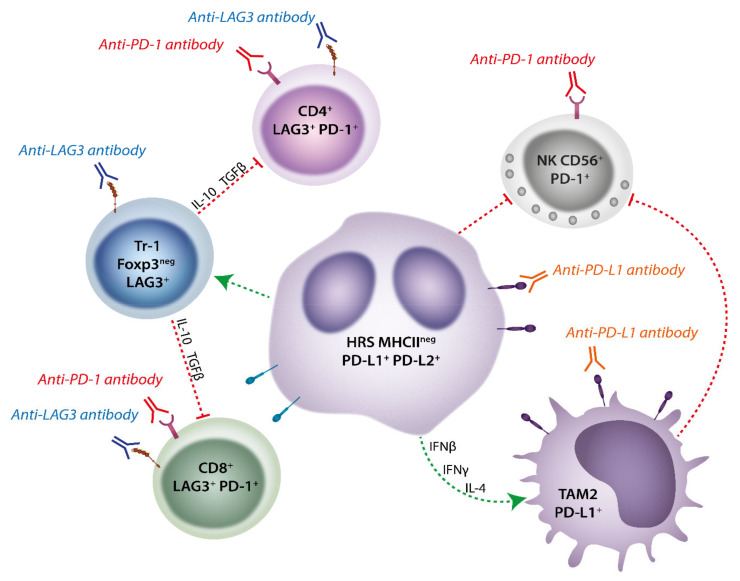
MHC-II Deficient Classical Hodgkin Lymphoma Fosters a Unique Tumor Microenvironment that is amenable to dual LAG-3/PD-1 blockade. In cHL cases MHC-II deficient HRS cells can form an immunotolerant environment by the enrichment of type 1 TREG (Tr1) cells that are defined by high expression of LAG-3 and lack of Foxp3. These cells secrete immunosuppressive cytokines (i.e., IL-10 and TGFβ) that contribute to the exhaustion of both CD8^+^ and CD4^+^ effector T-cells. PD-L1 is highly expressed on HRS cells (driven by 9p24.1 genomic alterations as well as TAMs which are skewed towards the PD-L1-expressing M2 phenotype by HRS cells secretion of inflammatory cytokines (IL-4, INFγ, INFβ). This high expression of PD-1 ligands in the microenvironment further inhibit effector T-cell function as well as CD56+NK cells, which are integral immune effectors in MHC deficient tumors. Application of anti-LAG-3 and/or anti-PD-1 antibodies can inhibit Tr-1 cells and disinhibit effector NK, CD4^+^, or CD8^+^ cells, whereas anti-PD-L1 blockade targets TAMs and HRS cells. By targeting different cell populations, anti-LAG-3 and anti-PD-1 directed therapy may be a powerful combination to reinstate immune control in cHL that lacks MHC-II expression.

A recent immunohistochemistry (IHC) based study has shown that LAG-3 is infrequently expressed in HRS cells but is commonly found in the surrounding immune infiltrating cells and appears to be higher in regions adjacent to the malignant cell [72]. This is in contrast to PD-L1 which is expressed in the vast majority of malignant HRS cells in cHL. Many infiltrating effector TILs in the TME in this study expressed TIM-3 and PD-1 as well as LAG-3 indicating the complex interactions involving multiple immune checkpoints that exists to support the HRS cells. Recently, Aoki et.al have demonstrated in a seminal study a TME enrichment with type 1 T_REG_ (Tr-1) cells defined by their high expression of LAG-3 that were absent in reactive lymph nodes using single cell RNA sequencing [73]. These cells co-expressed IL-10 and TGFβ and were demonstrated to be immunosuppressive. In addition, the authors demonstrated that the significant immunosuppressive effect on T-cells from cHL patients could be reversed on removing the LAG-3 expressing Tr-1 cells. Interestingly, these cells appeared to be spatially located close to HRS tumor cells with loss of MHC-II, a likely key mechanism of evasion of anti-tumor CD4^+^ effector T-cells in cHL. This contrasted with conventional (FOXP3^+^LAG-3^−^) T_REG_ cells which appeared to more closely associate with MHC-II expressing HRS tumor cells. While LAG-3 was expressed on Tr-1, PD-1 was not commonly found on these cells, suggesting a hypothetical benefit of combination anti-PD-1 and anti-LAG-3 blockade in cHL by potentially targeting very different T-cell populations in the TME (Figure 3). Supporting the role of LAG-3 expressing cells contributing to the development of a permissive TME in cHL, a clinical cohort were described in which high numbers of LAG-3^+^ TILs within the diagnostic biopsy showed a trend for inferior outcome when treated with ABVD (Doxorubicin, Bleomycin, Vinblastine, and Dacarbazine) chemotherapy.

In further recent work, Nagasaki et al. has also highlighted the important role of LAG-3 in inhibiting CD4^+^ anti-tumor responses in cHL [74]. In particular, CD4^+^ responses to anti-PD-1 were critical in the setting of MHC-I deficient tumor models where MHC-II expression remained intact. Anti-PD-1 therapy augmented these CD4^+^ responses and demonstrated a strong rationale for combination LAG-3/PD-1 based therapy to enhance both MHC-I and MHC-II responses. We have also recently demonstrated the importance of LAG-3 in cHL by showing that gene expression of LAG-3 was threefold higher in cHL compared to diffuse large B-cell lymphoma (DLBCL) cases [75]. This study also found that circulating LAG-3 is significantly elevated at diagnosis in cHL and decreases significantly with therapy.

In summary, the expression of LAG-3 amongst unique TIL subsets such as Tr1 cells, particularly in tumors with low MHC-II expression, may be critical to the development of a tolerogenic immune environment in cHL. This emerging work in cHL indicates promise for a combined immune checkpoint therapy utilizing anti-LAG-3 for cHL which is the current focus of actively recruiting clinical trials (NCI trial ID 02061761).

### 4.3. Immune Checkpoint Blockade Therapy in Classical Hodgkin Lymphoma

Amongst all LPDs, patients with cHL have demonstrated the best responses to PD-1 axis inhibition (Table 1). An early study of 23 R/R cHL patients, demonstrated objective response rate (ORR) of 87% and complete response rate (CR) of 17% to the PD-1 monoclonal antibody (mAb) nivolumab [76]. This excellent response was further demonstrated in the CheckMate 205 trial, including 80 patients with cHL who had previously undergone autologous stem cell transplant (ASCT) +/− treatment with brentuximab and achieved ORR of 71% and CR of 21% [77]. There have been similar results using alternative PD-1 mAb pembrolizumab, best demonstrated by the KEYNOTE-087 phase 2 trial which included patients who had either failed or were ineligible for ASCT +/− brentuximab vedotin or salvage chemotherapy showed an ORR of 71% and CR of 27.6% with a 3 year OS of 86% [78]. Additionally, in a recent phase III randomized controlled trial, pembrolizumab outperformed brentuximab in the post-ASCT R/R setting demonstrating a significantly longer PFS [79]. Overall, PD-1 blockade in R/R cHL is associated with high response rates and durable effects with acceptable safety profiles. Ongoing trials are assessing the utility of these molecules in the frontline setting.

PD-L1 expression is predictive of response to PD-1 checkpoint inhibition in a range of solid tumors [91,92]. In cHL, both CheckMate-205 and KEYNOTE-087 trials showed high levels of PD-L1 expression in cHL tumor cells [80,84] and higher levels of 9p24.1 copy gains were associated with improved PFS [93]. Correlative biomarker studies demonstrated PD-L1 expression on malignant HRS cells but not PD-L1 on infiltrating immune cells, was associated with treatment response [80].

PD-1 blockade is conventionally thought to increase the activity of CD8+ cytotoxic T-cells which interact with tumor antigen via MHC-I, however it has been found that HRS cells do not express MHC-I [94,95] suggesting anti-tumor immunity could be mediated by CD4+ effector cells via MHC-II antigen presentation [94]. Retention of MHC-II expression appears critical to anti-PD-1 response which is of particular relevance in cHL where >90% of cases have either absence of B2M or loss of MHC-I.

Studies of solid tumors, particularly melanoma, demonstrate that PD-1 blockade effects appear to be mainly attributable to the reinvigoration and clonal expansion of exhausted cytotoxic CD8+ T-cells. However, HRS cells are frequently MHC-I deficient, generating interest in the possibility of non-CD8+ T-cell-mediated mechanisms underpinning the efficacy of PD-1 blockade [96]. A recent study by Reinke et al. indicates that clearance of HRS cells from the TME following PD-1 blockade in the frontline setting did not involve T-cell expansion or increase in clonality and rather the early response was associated with the early clearance of HRS cells as well as local CD4^+^LAG-3^+^ TILs (i.e., Tr-1) and PD-L1+ TAMs. Thus, the clinical responses to anti-PD-1 therapy appear to be mediated by the loss of pro-survival signals in the TME rather than the promotion of a cytotoxic T-effector response [97].

This is contrasted with the effect of PD-1 blockade in the R/R setting where PD-1 blockade does demonstrate CD4+ clonal expansion that correlated with depth of response. This was demonstrated in a recent study by Cader et. al. who found that in comparison to healthy donors, patients with newly diagnosed cHL had a reduced TCR repertoire and this was reduced even further in patients with R/R disease [96]. In patients with R/R who received nivolumab, total and CD4^+^ specific TCR diversity at baseline and during therapy were associated with treatment response [94]. That is, PD-1 blockade was more effective in patients who had a diverse peripheral TCR repertoire. This finding is consistent with previous studies in solid malignancies which have found that recruitment of new T-cell clones, rather than expansions of previously identified tumor specific T-cells, was associated with a response to PD-1 inhibition [98]. In patients with the best response to PD-1 blockade, there was a significant increase in CD4^+^, but not CD8^+^ TCR clonal diversity. Further examination with a response to PD-1 inhibition included increased circulating activated NK-cells with a subset of cells which were CD3^−^/CD68^+^/CD4^+^/GrB^+^ which were also detected in the TME. This suggests a complimentary role of clonally diverse CD4^+^ T-cells and innate effectors in responding to PD-1 blockade [94]. This raises the intriguing possibility that in the R/R setting, cHL subclones may emerge that possess separate mechanisms of immunological escape although this finding is yet to be validated independently.

## 5. Checkpoint Molecules in Non-Hodgkin Lymphoma

### 5.1. Immune Checkpoint Molecules in Primary Mediastinal B-Cell Lymphoma

PMBCL is distinct from other B-NHL subtypes demonstrating clinical, morphological, and molecular features shared with cHL [99]. The genetic hallmarks of PMBCL are copy number alterations or translocations of the PDCDLG1 and PDCDLG2 genes (encoding PD-L1 and PD-L2, respectively) at locus 9p24.1 which are present in 60–70% of cases [51,100]. These genomic alterations occur at significantly higher frequency in PMBCL than other B-NHL subtypes. Accordingly, this correlates with increased PD-1 ligand expression on tumor cells [58,101,102]. Translocations of the 9p locus are highly specific for PMBCL often involving PDCDLG2 (gene encoding PD-L2) and lead to expression of PD-L2 at higher levels than PD-L1, a phenomenon not seen in other B-LPDs, including cHL [56,100,101,103]. MHC class II transactivator (CIITA), is a recurrent gene fusion partner for 9p.24 translocations in PMBCL which further reduced tumor immunogenicity through decreased antigen presentation and these translocations are associated with poorer outcomes [102].

PMBCL has recently been described to have high expression of LAG-3 within the TME at similar levels to that found in cHL. However, in this study, the authors found the vast majority of T-cells in PMBCL with LAG-3 expression were on CD8^+^ TILs [104] in contrast to cHL where CD4^+^ TILs appeared to be the predominant LAG-3 expressing T-cell [74]. Data regarding the functional status of these TILs remain sparse and further description of the co-expression of other inhibitory molecules in this NHL subtype are needed.

### 5.2. Immune Checkpoint Molecules in Primary Central Nervous System and Testicular Lymphoma

Primary CNS (PCNSL) and primary testicular lymphoma (PTL) present in areas of ‘immune privilege’. Like PMBCL, more than half of PCSNL/PTL cases have genomic alterations of 9p24.1 that result in constitutive PD-1 ligand expression on tumoral cells [105]. Additional molecular drivers of the pathogenesis of PCNSL/PTL include gain-of-function MYD88 mutations (65% of cases) and loss of MHC I and II molecules (50% of PCNSL and 80% of PTL), both of which are independent of PD-1/PD-1 ligand expression [105,106]. 

Given the TME and PD-1 axis have a significant role in dictating treatment outcome in PCNSL/PTL they are promising prognostic biomarker candidates. As described above, PD-L1 is over-expressed in the ‘immune privileged’ TME by several distinct mechanisms. While the total PD-L1 and tumor cells-restricted PD-L1 expression appears to have no association with clinical outcome, a favorable outcome is observed in patients with high PD-L1 expression on TAMs in both PTL and PCSNL treated with conventional therapy [107,108]. In PTL, high PD-1 expression on TILs (CD4^+^ and CD8^+^) correlates strongly with intra-tumoral PD-L1^+^ TAMs and is also associated with improved outcomes [108,109]. By contrast, high PD-1^+^ TILs in PCSNL conveys a poor prognosis, potentially reflecting high levels of T-cell exhaustion, which is particularly enriched in the rare EBV^POS^ subset occurring in immunocompromised patients [110,111,112,113]. Gene expression and multiplex IHC studies of PCNSL have found that co-expression of other immune checkpoint molecules (i.e., LAG-3 and TIM-3) in the TME is more strongly associated with poor outcome than PD-1 alone [109,114]. This implies that multiple markers to define states of T-cell exhaustion may be more valuable as a prognostic biomarker than PD-1 alone.

As seen in some cases of cHL, EBV is involved in lymphomagenesis through activation of the JAK/STAT pathway and transcription factor AP-1 [115]. EBV^POS^ PCNSL represents a rare but distinct subset of patients typified by unique immunobiology and poorer clinical outcomes [116]. Unlike the EBV^NEG^ counterparts, EBV^POS^ PCNSL seldom demonstrate increased rates of genomic alterations of 9p24.1 that could increase constitutional expression of PD-1 ligands [117]. Despite this, PD-L1 gene expression is several fold higher in EBV^POS^ cases which are also enriched for expression of LAG-3 and CD163 [108,118,119]. These findings are consistent with other EBV-infected LPDs including EBV^POS^ cHL [118,120], post-transplant lymphoproliferative disease, and plasmablastic lymphoma [13,119]. Further IHC studies have demonstrated that the majority of PD-L1/PD-L2 expression in EBV^POS^ PCSNL appears to be on microenvironmental cells, most notably TAMs, which co-expressed high PD-L1 and PD-L2 [106,107,108,116,121]. This is associated with significant T-cell exhaustion of intra-tumoral T-cells that co-express PD-1 along with other checkpoint molecules, LAG-3 and TIM-3 [116]. As such, EBV^POS^ lymphoma represent an attractive entity for trials of dual-checkpoint blockade to reinvigorate the intra-tumoral immune response.

Together, these findings indicate that ‘immune privilege’ is conferred through a variety of mechanisms in PCSNL and PTL. EBV^NEG^ tumors are dependent on genetically mediated immune evasion including 9p24.1 gains or translocations and loss of HLA-I/II loci whereas immune evasion in EBV^POS^ PCSNL is orchestrated by up-regulation of PD-L1^+^ M2 monocyte/macrophages along with LAG-3 upregulation and subsequent T-cell exhaustion.

## 6. Diffuse Large B-Cell Lymphoma

### 6.1. Role of PD-1 Axis in Diffuse Large B Cell Lymphoma

In DLBCL, the degree of PD-1 expression is highly heterogenous but generally demonstrates relatively low PD-1 expression on TILs compared with cHL and other B-NHL subtypes (such as PMBCL) [122]. Exceptions exist in certain histological subtypes of DLBCL which are consistently enriched with PD-1 expression, including germinal center B-cell (GCB) DLBCL and T-cell/histocyte-rich large B-cell lymphoma (THRLBCL) [122,123,124]. PD-1 ligands are seldom expressed on DLBCL cell lines in-vitro but have been demonstrated on malignant B-cells and TAMs in vivo suggesting PD-L1/2 expression is driven largely by tumor extrinsic, rather than intrinsic mechanisms [125]. The degree of PD-L1/2 expression is highly variable in DLBCL, perhaps reflecting the heterogeneity of the underlying pathobiology. Genomic gains or amplifications of 9p24.1 which drive PD-1 ligand expression in other B-LPDs are identified in a minority of DLBCL patients (10–15%) [126,127,128]. It has recently been recognized that EBV infection drives PD-L1 expression in DLBCL (<5% cases). In addition to increased PD-1 ligand expression, these EBV-driven DLBCL cases promote a highly immunotolerant TME with upregulated LAG-3, TIM-3 and immunosuppressive TAMs. Subsequently, EBV^+^ DLBCL patients experience worse clinical outcomes when treated with immunochemotherapy compared with EBV^-^ cases [129].

More recently, a unique genetic mechanism of immune escape caused by disruption of the 3′ untranslated region (UTR) of the PD-L1 gene has been demonstrated in DLBCL which led to a marked elevation of aberrant PD-L1 transcripts that are stabilized by truncation of the 3′ UTR [126]. PD-L1/PD-L2 expression is also regulated by the expression of microRNAs (miRNAs) [84,127,128,130,131]. These miRNAs either directly regulate PD-1 ligands expression through binding to the 3′ UTR of the PD-L1 or PD-L2 mRNA or indirectly by influencing the expression of PD-L1/PD-L2 regulators. In DLBCL, an inverse correlation was observed between expression of miRNAs, e.g., miR-195 or miR-214 and PD-L1. In-vitro experiments demonstrated that both miR-195 and miR-214 can target PD-L1 to reduce its expression. While miR-195 regulated immune responses of DLBCL through targeting PD-L1, miR-214 inhibited the tumor growth [132,133].

Unlike related NHL subtypes PMBCL and PCSNL, DLBCL PD-1 ligand expression is highly heterogenous and largely mediated by tumor-extrinsic mechanism. The multitude of mechanistic drivers of PD-1 ligand expression in DLBCL emphasizes the complexities of interpreting the TME for utility in prognostic or predictive biomarkers.

### 6.2. Role of LAG-3 in Diffuse Large B Cell Lymphoma

Data from the Cancer Genome Atlas (TCGA) shows that LAG-3 expression is highest in DLBCL compared to all other tumor types. The same study also demonstrated that LAG-3 correlated strongly with PD-L1 and CD8 expression and also showed association with high tumor mutation burden and viral expression and, in particular EBV, across all tumors [134].

Chen et al. showed relatively low expression of LAG-3 in DLBCL tumor cells in contrast to TIM-3 where 39% of malignant cells showed expression [135]. However, LAG-3 was expressed in the vast majority of TILs and dual blockade of LAG-3 and TIM-3 showed potent anti-DLBCL T-cell based immune killing. Anti-LAG-3 blockade also showed strong activity in cultured T-cells directed against DLBCL cell lines [135].

We have recently described high levels of LAG-3 in the tumor biopsy of patients as assessed by digital gene expression from a large cohort of newly diagnosed DLBCL cases (receiving standard chemo-immunotherapy) that was associated with inferior PFS and OS independently of conventional prognosticators [136]. In particular, tumors with high expression of both PD-L1 and LAG-3 appeared to be associated with particularly poor outcome. Another important finding from our study was that in DLBCL, LAG-3 is expressed by both malignant and non-malignant cells in the TME in a subset of patients. These results are consistent with previous studies which have reported LAG-3 expression by malignant cells in DLBCL using IHC [124,135]}. In addition, we investigated the expression of LAG-3 on TILs demonstrating its expression was highest on CD4^+^ T_REG_ cells but was also highly expressed on CD8^+^ T-cells compared to CD4^+^ non-T_REG_ cells. Importantly, LAG-3^+^ TILs were enriched in PD-1 and TIM-3 suggesting the potential for synergistic efficacy in combined immune checkpoint blockade.

### 6.3. Immune Checkpoint Molecules in Follicular Lymphoma

In follicular lymphoma (FL), TILs are enriched for ‘activated’ and (CD4^+^CD45RO^+^) T-cell populations (without signs of exhaustion) compared with other subtypes of NHL and normal lymphoid tissue [137]. Broadly, these FL TILs have higher PD-1 expression and are less responsive to cytokine stimulation [137,138]. However, while inhibitory receptors are abundant within the FL TME the biological implications of checkpoint receptor expression on TIL subpopulations are highly heterogenous. FL TILs are frequently observed in the intra- and peri-follicular regions and appear less frequently in the interfollicular regions [137,138,139,140]. The spatial distribution appears to be linked to the TILs functional status and checkpoint receptor expression. Yang et al. demonstrated two functionally distinct CD4^+^ T-cell subpopulations in the FL TME. TILs with a follicular helper (T_FH_) phenotype (CD4^+^, CXCR5^+^, TIM3^−^) are seen within the FL follicle and were PD-1^Hi^. Unexpectedly, these PD-1^Hi^ cells retained the capacity to secrete IL-12 and supported B cell growth in-vitro. By contrast, a second population, predominantly in the interfollicular regions of PD-1^Low^ TILs had an ‘exhausted’ phenotype (CD4^+^, TIM3^+^, and OX40^−^). These TILs displayed reduced cytokine production and cell-signal transduction [138].

The surprising finding that the PD-1^Hi^ TILs were predominantly responsible for production of inflammatory cytokines in the FL TME suggested that these cells retained effector function and PD-1 expression alone was insufficient to identify T-cell exhaustion in the FL TME. A subsequent study demonstrated that LAG-3 expression occurred exclusively on PD-1^+^ T-cells and frequently co-expressed other checkpoint molecules, particularly TIM-3. In a cohort of 28 patients with FL, those with the highest expression of LAG-3 had inferior outcomes and this was particularly the case for patients with T-cells co-expressing PD-1 and LAG-3. The PD-1^+^LAG-3^+^ cells did appear to identify a particularly exhausted phenotype of TIL and resulted in substantially reduced capacity to produce cytokines and cytotoxic granules [141]. In addition, only combined anti-PD-1 and anti-LAG-3 antibodies led to significant reinvigoration of exhausted T-cells in-vitro. This suggest dual checkpoint blockade may be a viable therapeutic avenue in FL.

The expression of ligands of PD-1 are also highly variable in FL. Tumor cells seldom express PD-L1 or PD-L2 [58,142,143] and it is uncommon for genomic alterations that result in upregulated ligand expression to occur [100]. Concordantly, in previously unpublished data, our group demonstrates in cohort of 175 patients with FL there were no instances of 9p24.1 (locus harboring PD-L1/PD-L2) copy number alterations. Rather PD-1 ligands appear to be expressed on stromal and myeloid-derived cells in the TME, likely induced through increased intra-tumoral expression of pro-inflammatory cytokines, i.e., IFN, TNF, and IL-4 [144].

Given the degree of complexity of checkpoint molecule expression in FL, studies of checkpoint molecule expression as a prognostic biomarker in those treated with conventional immunochemotherapy have produced conflicting results. Several studies have demonstrated that high expression of PD-1^+^ cells, particularly when expressed in the intrafollicular compartment, are associated with improved PFS and OS [139,145], however this effect may be abrogated in the rituximab-era [140,146,147,148]. Low PD-1 expression by IHC is also associated with high risk clinical features including high serum B2M [148] and grade III disease [145]. By contrast, recent study presented in abstract form has demonstrated that high levels of CD8^+^LAG-3^+^ cells in the TME of FL indicated a significantly higher risk for large cell transformation [142,145]. This association with transformed large cell disease in FL may indicate a significant role for LAG-3 related immune suppression in these diseases and warrants further study.

Recently, we have demonstrated that the FL TME can be dichotomized by gene-expression profiling of multiple immune markers into high- (hot) or low- (cold) immune infiltration states. This reveals significant enrichment for early progression of disease in the immunologically ‘cold’ tumors [144]. Low PD-L2 expression was an effective surrogate from a cold TME and correlated with low PD-1 and LAG-3 gene expression. Cold FL also demonstrated reduced inflammatory cytokine expression, lower T-cell infiltration, and reduced T-cell receptor clonality. This multi-omics approach reflects the coordinated expression of multiple inhibitory receptors and their ligands suggesting that a loss of immunogenicity may underpin the poor prognostic association with low checkpoint expression. As such, measures of immunogenicity may serve as more robust biomarkers in FL than the expression of any individual immune checkpoints or TIL subsets.

## 7. Immune Checkpoint Blockade Therapy in Non-Hodgkin Lymphoma

The high response rates to checkpoint inhibition seen in cHL have unfortunately not been replicated in patients with NHL, particularly in DLBCL (Table 2). An early phase 2 trial of nivolumab in R/R DLBCL showed an ORR of 3% in transplant-ineligible patients and 10% in patients who had relapsed after autologous transplant [143]. Likewise, the use of pembrolizumab as consolidation post-ASCT did not seem to add a therapeutic benefit [149].

Interestingly immune-checkpoint inhibition may play a role in priming the immune system to another targeted monoclonal antibody therapy. Although poor outcomes were seen in a recent study of single-agent nivolumab in FL showing a 4% response rate [157], in an interim analysis of a phase 2 study of treatment naïve patients with FL treated with nivolumab and rituximab showed more promising results with an ORR of 84% and a CR of 47% was reported. This suggests adjuvant approaches to improve immunogenicity may sensitize FL to ICB therapy [158].

In keeping with the frequent alterations of chromosome 9p24.1 and PD-L1/PD-L2 expression seen in certain NHL subtypes, PMBCL, PCNSL, and PTL studies have shown more favorable responses to PD-1 blockade. A subset of patients with R/R PMBCL in the phase 2 KEYNOTE-013 study had an ORR of 48% and CR of 33% with median duration of response not reached after a median follow up of 29 months [86,150]. Further benefit of combination therapy in R/R PMBCL with novel agent brentuximab appear promising as evidenced by the CheckMate 436 study which showed excellent ORR of 70% and CR rate of 43%. A small study has also been conducted in R/R PCNSL or PTL that showed PD-1 blockade resulted in ORR of 100% with prolonged duration of response [154], although larger phase II studies in R/R PCNSL recently presented in abstract form have since demonstrated less promising results [153].

The efficacy of checkpoint inhibition in mature B-NHL subtypes, particularly DLBCL and FL, is limited. Some factors are associated with better clinical responses. Gene alterations of *PD-L1* are found in ~15% of DLBCL patients and is more frequently observed in non-GCB subtype [68,159]. This subset of patients have a better response to PD-1 blockade [159] in keeping with other subsets of NHL that frequently harbor genetic alterations of chromosome 9p24.1. Furthermore, a study of relapsed NHL compared the efficacy of pembrolizumab in EBV^POS^ and EBV^NEG^ showed an increased response rate and higher PD-L1 expression in EBV^POS^ tumors [160].

These results demonstrate that the use of current checkpoint blockade therapy may be best reserved for lymphoma subtypes with genomic alterations that promote high levels of PD-L1/PD-L2 expression (i.e., cHL, PMBCL, PCNSL, and PTL). While the low response rates in other lymphoma subtypes have been underwhelming, further clinical trials are warranted to determine whether other subtypes of NHL responses to PD-1 blockade can be improved through the combination with immunogenic anti-CD-20 monoclonal antibodies or dual checkpoint inhibition.

There is still limited single agent data for the use of anti-LAG-3 based therapy in lymphoma. In a small series of NHL treated in a phase I study, there was minimal response to therapy indicating that this agent may need to be combined with other agents to elicit responses [161].

## 8. Future Directions

Both PD-1 and LAG-3 represent emerging mechanisms of immune escape in LPD and are promising targets for therapeutic intervention. Pre-clinical studies suggest the synergistic role of dual blockade of these pathways may be more efficacious than either strategy alone due to improved re-activation of exhausted effector TILs as evidenced in DLBCL or by targeting separate populations in the TME as evidenced in cHL. Additionally, combinations of single or dual ICB therapy with sensitizing agents that promote immunogenic cell death (i.e., radiotherapy, immune vaccines, and oncolytic viruses) are hypothesized to improve tumor immunogenicity may broaden the cohort of patients that are responsive to immunotherapy as suggested by recent developments in FL.

As well as opportunities to enhance immunogenicity, manipulation of the PD-1 and LAG3 axis also show promise as a strategy to improve responses to adoptive T-cell therapies such as chimeric antigen receptor T-cells (CAR-T). Studies using CRISPR-Cas9 mediated gene editing demonstrate that the knockout of PD-1 and LAG3 in CAR-T cells overcome the immunosuppressive nature of the tumor environment, a key factor limiting CAR-T efficacy [162,163,164,165]. As such, the outcomes of current clinical studies of dual checkpoint blockade and associated translational studies in lymphoproliferative disease are eagerly awaited.

## Figures and Tables

**Table 1:** Pivotal Trials in the Use of Immune Checkpoint Blockade in Classical Hodgkin Lymphoma.

Clinical Trial (Year)	Pt Characteristics	Experimental Arm	Study	ORR	CR Rate (%)	PFS, OS, DOR	Grade 3–5 AEs	Ref.
**R/R cHL Trials**								
CheckMate 039 (2015)	R/R cHL	Nivolumab 3 mg/kg q2 weekly CR, PD or excessive toxicity	Phase 1 23 pts	87%	17%	PFS at 24 weeks = 86%	22%	[76]
CheckMate 205 (2016–2018)	R/R cHL, Previous ASCT Pt Subsets: A: BV Naïve B: Prev BV after ASCT C: Prev BV before +/− after ASCT	Nivolumab 3 mg/kg q2 weekly until PD excessive toxicity	Phase 2 243 pts A = 63 B = 80 C = 100	Overall 71% A = 65% B = 71% C = 75%	Overall 21% A = 32% B = 14% C = 20%	Median PFS = 15 months Median OS = Not reached Median DOR = 18 months	40% ^1^	[77,80]
NCT 03004833 (2017–2019)	R/R cHL, No prior ASCT	BV 1.8 mg/kg + Nivolumab 3 mg/kg q3 weekly × 4 cycles +/− ASCT	Phase 1/2 91 pts	85% 74% proceed to ASCT	67%	PFS at 24 months = 78% ^2^ OS at 24 months = 93%	31%	[81,82]
ECOG-ACRIN Research Group NCT01896999 (2020)	R/R cHL Included previous allogeneic (*n* = 8) and autologous (*n* = 21) transplant patients	3 Experimental Arms: A = BV (1.8 mg/kg) + Ipilimumab (1 mg/kg or 3 mg/kg) B = BV (1.2 mg/kg or 1.8 mg/kg) + Nivolumab (3 mg/kg) C = BV (1.2 mg/kg) + Ipilimumab (1 mg/kg) + Nivolumab (3 mg/kg)	Phase 1/2 61 pts A = 21 B = 18 C = 22	A = 76% B = 89% C = 82%	A = 57% B = 61% C = 73%	A: Median PFS = 14.4 months B: Median PFS = Not reached C: Median PFS = Not reached	A = 43% B = 16% C = 50%	[83]
KEYNOTE-087 (2017–2019)	R/R cHL Pt Subsets: A: BV Naïve, Previous ASCT B: Previous BV after ASCT C: Previous BV, no ASCT	Pembrolizumab (200 mg) q3 weekly for 2 years	Phase 2 210 pts A = 60 B = 69 C = 81	Overall 71% A = 71.7% B = 78.3% B = 64.2%	Overall 27.6% A = 31.7% B = 26.1% B = 25.9%	Overall: Median PFS 13.7 months Median DOR 16.6 months A: Median PFS 16.4 months B: Median PFS 11.1 months C: Median PFS 19.4 months A: Median DOR 22.1 months B: Median DOR 11.1 months C: Median DOR 24.2 months	11.9%	[84,85]
NCT02362997 (2019)	R/R cHL Consolidation post-ASCT	Pembrolizumab 200 mg 3 weekly × 8 cycles ^3^	Phase 2 30 pts	N/a	N/a	PFS at 18 months = 82% OS at 18 months = 100%	30%	[86]
KEYNOTE-013 (2020)	R/R cHL, Previous BV Previously failed or ineligible for ASCT	Pembrolizumab 10 mg/kg 2 weekly for 2 years	Phase 2 31 pts	58%	19%	Median PFS 11.4 months OS at 36 months = 81% DOR at 36 months = 50%	6%	[87]
**Frontline Hodgkin Lymphoma Trials**								
CheckMate 205 (2019)	Advanced-Stage cHL Treatment Naïve	Nivolumab (240 mg) q2 weekly × 4 cycles Followed by Nivolumab + AVD q2 weekly × 12 cycles	Phase 2 51 pts	75%	84%	PFS at 21 month 80%	59%	[88,89]
GHSG NIVAHL 2020	Early-Stage Unfavorable cHL Treatment Naïve	2 Experimental Arms ^4^: A = Nivolumab (240 mg) + AVD × 4 cycles B = Nivolumab q2 weekly for 2 cycles followed by Nivolumab-AVD × 2 cycles followed by AVD × 2 cycles.	Phase 2 109 pts A = 55 B = 54	A = 100 B = 98	A = 83 B = 84	PFS at 12 months = 99% OS at 12 months = 100%	A = 28% B = 38%	[90]

ASCT = Autologous Stem Cell Transplant, AVD = Adriamycin, Vinblastine, Dacarbazine, BV = Brentuximab Vedotin, DOR = duration of response, OS = Overall Survival, PFS = Progression Free Survival. ^1^ Adverse event figures derived from the post ASCT + Brentuximab vedotin cohort. ^2^ 24-month PFS 91% for pts who underwent ASCT following experimental arm. ^3^ Received anti-PD-1 as consolidation therapy following autologous stem cell transplant. ^4^ Both experimental arms received 30 Gy involved site radiotherapy following treatment.

**Table 2:** Pivotal Trials in the Use of Immune Checkpoint Blockade in Non Hodgkin Lymphoma.

Clinical Trial (Year)	Pt Characteristics	Experimental Arm	Study	ORR	CR Rate (%)	PFS, OS, DOR	Grade 3–5 AEs	Ref.
DLBCL Trials								
NCT02038933	R/R DLBCL Pt Subsets: A: Previously Failed ASCT B: Ineligible for ASCT	Nivolumab (3 mg/kg) q2 weekly	Phase 2 121 pts	A = 10% B = 3%	N/a	A: Median PFS = 1.9 months B: Median PFS 1.4 months	20%	[143]
NCT02362997 (2019)	R/R DLBCL Following ASCT ^1^	Pembrolizumab 200 mg q3 weekly × 8 cycles.	Phase 2 29 pts	N/a	N/a	PFS at 18 months = 59% ^2^	79%	[149]
PMBCL Trials								
KEYNOTE-013 (2017)	R/R PMBCL	Pembrolizumab 200 mg q3 weekly for 2 years or until toxicity or PD	Phase 1 21 pts	48%	33%	Median PFS = 10.4 months Median OS = 31.4 months Median DOR not reached	24%	[87,150]
CheckMate 436 (2019)	R/R PMBCL	Nivolumab (240 mg) + BV q3 weekly	Phase 2 30 pts	70%	43%	Median PFS = not reached Median OS = not reached	53%	[151]
KEYNOTE 170 (2019)	R/R PMBCL	Pembrolizumab 200 mg q3 weekly for 2 years (or until PD toxicity or patient withdrawal	Phase 2 53 pts	45%	21%	Median PFS = 5.5 months Median OS = not reached Median DOR = not reached	23%	[152]
Other NHL Trials								
Acsé Trial (2020)	R/R PCNSL	Pembrolizumab 200 mg q3 weekly for 2 years	Phase 2 50 pts	26%	16%	Median PFS = 2.6 months Median DOR = 10.0 months	10%	[153]
DFCI Case Series (2017)	R/R PCNSL and PTL	Nivolumab 3 mg/kg q2 weekly	Case Series 5 pts	10%	80%	PFS at 17 months = 60% OS at 17 months= 100%	20%	[154]
NCT01592370 (2016)	R/R NHL and Myeloma Pt Subsets: A: FL B: DLBCL C: Other B-NHL	Nivolumab 1 mg/kg then 3 mg/kg	Phase 1 B-NHL 31 pts A = 10 pts B = 11 pts C = 10 pts	A = 40% B = 36% C = 0%	N/a	N/a	21%	[155]
NCT00904722 (2014)	R/R FL	Rituximab 375 mg/m2 weekly × 4 cycles + Pidilizumab 3 mg/kg q4 weekly × 12 cycles ^3^	Phase 2 32 pts	66%	52%	Median PFS = 18.8 months Median DOR = 20.2 months	0%	[156]
CheckMate 140 (2020)	R/R FL	Nivolumab 3 mg/kg q2 weekly	Phase 2 92 pts	1%	4%	Median PFS 2.2 months Median DOR 11 months	49%	[157]
1st FLOR (2019)	Frontline FL Treatment Naïve	Nivolumab (240 mg) q2 weekly × 8 cycles + Rituximab 375 mg/m^2^ 2 weekly × 4 cycles Followed by Nivolumab maintenance q4 weekly × 12 cycles + Rituximab maintenance q12 weekly × 8 cycles	Phase 2 39 pts	84%	37%	N/A	15%	[158]

B-NHL = B-cell Non Hodgkin Lymphoma, DOR = duration of response, OS = Overall Survival, PFS = Progression-Free Survival. **^1^** Consolidation following autologous stem cell transplant, 86% of pts were in CR at study baseline. ^2^ Primary endpoints were improving PFS at 18 months post ASCT from 60% to 80%. PFS at 18 months was 59% therefore did not meet primary end point. ^3^ Cycles 5–12 optional infusions q4 weekly if stable disease or better following first 4 cycles.
